# Supplementary material for: Are carbon emissions trading and green financial instruments synergistic? -Comprehensive quantitative research based on content analysis
Source: PLoS One. 2024 Mar 7;19(3):e0298601. doi: 10.1371/journal.pone.0298601 (PMC10919601; doi:10.1371/journal.pone.0298601)
Supplement: S1 Data — (ZIP) [file pone.0298601.s001.zip › Data/地级市Z_F工作报告.zip!/░▓╗╒/┘±╓▌╩╨/└┤╫╘╣1⁄2╓┌║┼í╛┬φ┐╦╩2╛▌═°í┐_2011.docx]

各位代表：
　　 现在，我代表亳州市人民政府，向大会报告政府工作，请予审议，并请市政协委员和其他列席人员提出意见。
　　 
　　 一、过去五年，励精图治、成果丰硕
　　 
　　 过去五年，是亳州发展史上极不平凡的五年。我们既经历了国际金融危机、多重自然灾害等重大考验，也迎来了中央扩大内需、促进中部地区崛起和省委、省政府加快皖北地区发展等难得机遇。在市委的坚强领导下，市政府全面贯彻落实科学发展观，紧紧依靠全市人民，坚持“发展为上、民生为先、稳定为重，少说多做、真抓实干”，解放思想，抢抓机遇，迎难而上，奋力拼搏，圆满完成市二届人大历次会议确定的目标任务，全市经济社会进入了加快发展的新阶段。
　　 ——五年来，我们始终坚持以经济建设为中心，着力调整经济结构，统筹发展三次产业，综合实力明显跃升。
　　 预计，2010年实现生产总值505亿元，是“十五”末的2。1倍，年均增长12。2%，三次产业结构由“十五”末的32：27。5：40。5调整为26。7：37。6：35。7；主要经济指标保持了两位数增长，实现了工业增加值、固定资产投资、财政收入在2007年基础上翻一番的奋斗目标。2010年，完成财政收入42。37亿元，是“十五”末的3。8倍；财政支出突破100亿元，是“十五”末的4。4倍。2009年，我市荣获省政府目标管理考核第二名，蒙城县、涡阳县被评为全省科学发展先进县。
　　 大力实施“125”行动计划和“工业经济三年倍增计划”，工业化水平显著提高。“十一五”期间，医药制造、煤炭开采和洗选、农副食品加工、交通运输设备制造业增加值，年均增长35%以上，主导产业不断发展壮大。2010年，全市规模以上工业实现增加值115亿元，是“十五”末的4。1倍；全市规模企业达到552家，比“十五”末增加401家；销售收入超亿元的企业由19家发展到70家；荣获中国驰名商标2件、省著名商标57件。全市开发区已建成面积32平方公里，比5年前扩大10倍；市南部新区工业组团入驻项目45个，现代中药产业园一期工程即将竣工。县域工业经济实力明显增强，非公有制经济健康发展，节能减排目标全部实现。
　　 全面落实各项强农惠农政策，产业化步伐明显加快，农业和农村经济持续发展。累计兑付粮食直补、农机补贴等财政性补贴资金34亿元。大力实施粮食增产工程、畜牧振兴计划和农业产业化“532”提升行动，粮食总产达92亿斤，比“十五”末增长44。4%，连续5年受到省政府表彰；涡阳县率先实现小麦亩产千斤县目标，连续两年荣获“全国粮食生产先进县标兵”称号。全市中药材、蔬菜种植面积、活立木蓄积量较“十五”末分别增长45%、42%、40%，规模养殖比重提高25个百分点，特色产业规模不断扩大。农业产业化龙头组织突破800家，农产品流通体系进一步完善。劳务经济加快发展，累计转移农村富余劳动力600万人次。农业装备水平不断提高，其中农业机械总动力达到680万千瓦，比“十五”末增长33。5%。
　　 城乡消费市场繁荣活跃，第三产业快速发展。预计，2010年，实现社会消费品零售总额227亿元，是“十五”末的2。3倍。苏果超市等国内知名商贸企业落户亳州，电子商务、连锁经营、物流配送等新型业态快速发展，商贸服务业改造升级步伐加快。大力推进“万村千乡”等市场工程建设，新建、改建并通过验收农家店1913家，城乡市场体系逐步完善。深入实施“旅游业发展三年行动计划”，市博物馆、曹操纪念馆、涡河公园、魏武广场、宋汤河景观带建成开放，市北关历史街区改造一期工程全面完成，古井酒文化博览园、花戏楼景区被评为国家4A级景区。五年累计接待游客1387万人次，实现旅游总收入74亿元，分别是“十五”时期的2。5倍和2。9倍。四家国有商业银行全部升格为二级分行，邮储银行、药都银行成功组建，徽商银行落户亳州，10家小额贷款公司获批筹建；五年累计新增贷款140。9亿元，是“十五”期间的5。5倍，金融对地方经济社会发展的支持力度进一步加大。商业保险快速发展，政策性农业保险全面推进。
　　 ——五年来，我们始终坚持打基础、管长远不动摇，全力推进项目建设，城乡建设取得新突破，发展基础更加坚实。
　　 过去五年，全市基础设施建设投入力度之大、开工项目之多，超过了历史任何时期。共争取国家和省各类项目投资22。9亿元，比“十五”时期净增6。4亿元。组织实施重大项目692个，完成投资353亿元。五年累计完成固定资产投资717亿元，是“十五”时期的4。4倍，年均增长38%。
　　 交通、电力、信息工程建设力度加大。济广高速公路亳州段建成通车，宁洛高速公路亳州段改造、泗许高速公路亳州段主体工程如期完工，亳州火车站改扩建工程顺利完成，全市农村公路总里程由2005年的6500公里增加到1万公里以上。220千伏变电站增加到5座，110千伏变电站增加到14座，全市主变电容量2500兆伏安，是“十五”末的2。7倍。信息通讯基础设施逐步完善，覆盖范围和用户数量不断扩大。
　　 城镇基础设施建设全面推进。完成《亳州市城市总体规划》修编工作。先后实施了主城区道路、桥梁、出入口、人行道改造工程，完善了污水处理厂、垃圾处理场、污水管网等一批市政公用设施，改造城区供水管网520公里，铺设城市燃气管网220公里，主城区面积扩大到40平方公里，全市城镇化率提高到35%，城市综合服务功能显著增强。深入开展文明创建活动，建成区绿化覆盖率达35%，历史名城保护工作得到加强；强化城市综合执法管理，市容市貌明显改观，创建安徽省园林城市目标如期实现。市南部新区自2008年启动以来，累计拆迁46万平方米，征地15000多亩，完成基础设施投入30亿元，集聚辐射功能开始显现。三县县城面貌变化巨大，小城镇建设步伐不断加快。全面完成村镇建设规划编制任务，省市级新农村建设示范镇、村分别发展到18个、151个。蒙城县被评为全省首批文明县城、省级园林县城。
　　 农村基础设施建设继续加强。水利工程累计投资32。4亿元，是“十五”期间的7。8倍。投资13亿元的涡河近期治理工程顺利竣工，淮北大堤除险加固、茨淮新河灌区续建配套、阚疃泵站除险加固等重点工程相继建成并发挥效益，新增和改善有效灌溉面积201万亩、旱涝保收田面积152万亩，解决了农村107。8万人的安全饮水问题。新建户用沼气2。6万户，完成生态造林17。8万亩，森林覆盖率达到18%。
　　 ——五年来，我们始终注重体制机制创新，积极推进重点领域和关键环节的改革，全方位扩大对外开放，发展活力进一步增强。
　　 农村土地流转和复垦整理步伐加快，全市累计流转土地71万亩，通过复垦整理，共新增耕地6。4万亩；林权制度改革深入推进，确权到户面积25。5万亩；率先在全省实施为民服务全程代理制度，农村基本公共服务体系不断健全；新型农村社会养老保险试点成功，新型农村合作医疗全面实施，基层医药卫生体制改革全面推开。城市建设投融资管理机制逐步健全，经营性土地招拍挂、政府采购制度执行到位。城区管理体制进一步理顺，行政综合执法能力不断加强。国企改革继续深化，古井集团40%股权转让、双轮集团改制圆满完成。广播电视资源有效整合，文化体制改革走在全省前列。新一轮市政府机构改革全面完成，县区、乡镇机构改革进展顺利，事业单位改革有序推进。
　　 坚持招商是第一要事，强化宣传推介，优化投资环境，成功举办5届全国（亳州）中药材交易会、4届国际（亳州）中医药博览会、2届中国（亳州）华佗五禽戏养生健身节和2届中国（亳州）白酒文化节，积极组团参加国内外各类重大节会招商活动。五年来，累计引进市外内资650亿元，实际利用外资4。9亿美元，年均分别增长68%和40。3%。淮北矿业等四大煤业集团、江淮汽车、康美药业、重庆啤酒等一大批知名企业入驻亳州，对外开放开始向高层次、宽领域迈进。加强与中央和省属大企业、国内知名民营企业及芜湖市的对接交流，在产业配套、经贸往来和文化旅游合作等领域取得了实质性进展。对外贸易在困境中突破，新增海关A类管理企业11家，进出口总额由“十五”末的1846万美元提高到2010年的2。2亿美元，年均增长64。1%。对外交往日益密切，与6个国家的7个城市建立了友好关系，亳州在国内外的知名度和影响力进一步提高。
　　 ——五年来，我们始终坚持以人为本，着力保障和改善民生，加快发展社会事业，人民生活水平大幅提高。
　　 从2007年开始实施民生工程以来，逐年提标扩容，4年累计投入68亿元，惠及500多万城乡居民。全面落实义务教育“两免一补”、贫困寄宿生补助、粮食直补、农机具补贴等政策，人民群众享受到了发展带来的更多实惠。认真落实全民创业、促进就业各项政策，累计新增城镇就业岗位14万个，城镇登记失业率控制在4%以内。养老、医疗、失业、工伤、生育保险覆盖面不断扩大，保障水平明显提高。新建、改扩建农村敬老院120所，全市五保集中供养率达到35%，利辛县被评为“全国五保供养先进县”。城乡低保、医疗救助、救灾救济、慈善救助等工作稳步推进，廉租住房、经济适用住房和住房公积金工作成效显著。扶贫开发整村推进力度加大，7。5万农村贫困人口实现脱贫。2010年，城镇居民人均可支配收入达到15445元，农民人均纯收入达到4375元，五年年均分别增长14。1%和13。2%。
　　 社会事业全面发展。教育事业发展较快，以县为主的教育管理体制全面建立，义务教育经费保障机制进一步健全，高中阶段教育普及程度大幅提高，中等职业教育和学前教育快速发展，高等教育规模不断扩大；中小学校舍安全工程扎实推进，新建、加固、改造校舍395万平方米；特殊教育学校建设、“关爱留守儿童”工程等加快实施，亳州师专新校、亳州一中南校、亳州九中、亳州十二中建成使用，华佗技师学院开工建设，亳州职业技术学院成功通过国家人才培养工作评估验收。累计完成教育投入98亿元，是“十五”期间的2。72倍。科技进步与创新步伐加快，新增国家级创新试点企业1家、高新技术企业8家、安徽名牌产品30个，现代中药特色产业基地被列入科技部“火炬计划”。卫生事业蓬勃发展，覆盖城乡的公共卫生服务体系不断完善，市医院南院投入使用，新建44个乡镇卫生院、1143个村卫生室、80个社区卫生服务站，478万人参加新农合和城镇医保。文化事业和文化产业繁荣发展，全市新建32个乡镇综合文化站、619个农家书屋，涡阳老子文化生态园开工建设，“老子传说”、“二夹弦”被列入国家非物质文化遗产保护名录，谯城区梆剧团根据沈浩事迹编演的话剧《民声的印记》受到广泛好评。广播电视实现全覆盖，新闻出版工作取得新成绩。全民健身运动广泛开展，市体育场建成使用，242个农民体育健身工程项目全部完工，“五禽戏”进校园、进企业、进机关活动受到广泛欢迎；全市竞技体育水平有了新提高，涡阳县武术选手张军勇夺得第十六届亚运会65公斤级散打金牌。人口和计划生育工作成效显著，低生育水平基本保持稳定，出生人口性别比升高势头得到有效遏制。国土和矿产资源管理得到加强，土地市场进一步规范。价格调控和监管工作成效明显，第六次全国人口普查工作进展顺利，人民防空工作取得了新成绩。地方志办公室被授予“全国先进单位”称号。国税、地税、工商、质监、人事、编制、档案、外事、侨务、对台、港澳事务、科协、老龄、供销社、残疾人、地震、气象、无线电管理等工作都取得了新的进步。
　　 ——五年来，我们始终坚持依法治市，推进和谐社会建设，社会大局稳定、人民安居乐业的良好局面进一步巩固。
　　 加强“平安亳州”建设，全面建立应急管理体系，扎实推进社会治安综合治理，严厉打击各类违法犯罪活动，认真排查化解各种矛盾纠纷，重点开展专项治理活动，健全完善信访接待日制度，切实加强安全生产监管，信访工作和安全生产形势总体平稳，命案侦破工作连续5年位居全省前列，一些社会热点难点问题得到及时有效解决，社会大局和谐稳定。深入开展“五五”普法和法律“六进”活动，加强人民调解和法律援助工作，依法治市进程全面推进。国防教育、国防动员和后备力量建设得到加强。驻亳人民解放军、武警部队、预备役部队和民兵，在支持地方经济建设、抗灾抢险等方面作出了重要贡献。积极开展拥军优属、拥政爱民活动，军政军民关系更加密切，被授予“全省双拥模范城”荣誉称号。全面贯彻党的民族宗教政策，保持了民族团结、宗教和顺的良好局面。
　　 在加快发展的进程中，我们始终高度重视政府自身建设。扎实开展创先争优活动，进一步提高科学发展的能力和水平。认真执行《行政许可法》，规范和清理行政审批项目，加强行政复议工作，完善相对集中行政处罚权制度，政府依法行政能力明显提高。畅通与群众联系渠道，不断改进市民论坛、市长热线、行风热线、在线访谈工作，落实政务公开、政府信息公开、重大决策咨询、听证、新闻发布等制度，加快推广电子政务，政府工作透明度进一步增强。自觉接受人大法律监督、政协民主监督和社会舆论监督，坚持向人大及其常委会报告工作制度和规范性文件备案制度，注重听取各民主党派、工商联、党外人士的意见和建议，进一步密切与工会、共青团、妇联等人民团体的联系。五年来，共办理人大议案20件、代表建议574件，满意率达98%；办理政协委员提案1386件，满意率和基本满意率达95%。认真落实廉政建设责任制，加强审计监督和行政监察，公务员廉洁从政意识显著增强，良好的政治生态初步形成。
　　 各位代表！刚刚过去的2010年，是地级亳州市组建10周年，也是“十一五”的收官之年。我们以保增长、扩内需、强基础、调结构、促和谐为主线，全力打造园区、融资、环境三大发展平台，加快培育现代中药、能源化工、白酒及农产品加工、汽车及零部件制造、文化旅游等五大产业，大力推进产业、城乡、民生等各项建设，全市呈现出经济快速发展、民生持续改善、社会和谐稳定的良好局面，确保了“十一五”各项目标任务全面完成。与2009年相比，全市生产总值增长13%，财政收入增长37。06%，固定资产投资增长40%，规模以上工业增加值增长29%，社会消费品零售总额增长19%，进出口总额增长35。3%，外商直接投资增长48%。这一年，我们成功举办了庆祝地级亳州市组建十周年活动，极大地提振了全市人民加快发展的信心；扎实开展了“招商引资年”、“项目建设年”和“环境优化年”活动，组织实施重点项目261个，完成投资150亿元，极大地促进了经济社会又好又快发展；大力推进城市建设，在去年人代会上承诺的十大重点建设工程，除建安路涡河大桥和105国道涡河大桥工程因建设方案调整暂未开工外，其余工程均如期完成计划目标，极大地改善了人居环境；高度重视保障和改善民生，城镇居民人均可支配收入和农民人均纯收入均增长10%，31项民生工程全面完成，极大地提高了人民群众的生活水平。
　　 各位代表！
　　 过去五年，是地级亳州市组建以来经济发展最快、城乡面貌变化最大、人民群众得到实惠最多的时期，是科学发展观在亳州大地生动实践的一段光辉历程。五年来，全市广大干部群众风雨兼程，顽强拼搏，一步一个脚印，一年一大台阶，取得了丰硕的成果，谱写了壮丽的篇章，我们完全有理由为取得的成绩而感到骄傲和自豪！
　　 回顾五年来的工作，我们深刻体会到：要加速亳州崛起，必须坚持以解放思想为先导，与时俱进，开拓创新，积极探索符合亳州实际的发展道路；必须坚持以科学发展观为统领，统筹兼顾，协调推进，全力促进经济社会全面协调可持续发展；必须坚持深化改革、扩大开放，不断增强加快发展的动力与活力；必须坚持以人为本、富民为先，切实加强以保障和改善民生为重点的社会建设；必须坚持在市委的领导下，凝聚全市人民力量，形成万众一心、团结奋进的强大合力；必须坚持依法行政，转变作风，努力形成真抓实干的工作局面。
　　 各位代表！五年来的发展成就，五年来的宝贵经验，为我市“十二五”时期科学发展奠定了坚实基础。这些成就和经验的取得，是省委、省政府正确领导的结果，是历届市委、市人大、市政府、市政协锐意进取、埋头苦干的结果，是600万亳州儿女众志成城、奋发图强的结果！在此，我代表市人民政府，向全市各族人民，向广大工人、农民、知识分子、干部、驻亳解放军指战员、武警官兵和政法干警，向各民主党派、各人民团体和社会各界人士，向所有关心、支持和参与亳州建设的人们，表示衷心的感谢并致以崇高的敬意！
　　 在总结过去五年工作、充分肯定成绩的同时，我们也清醒地看到，我市经济社会发展中还面临着一些不容忽视的矛盾和问题。主要是：经济总量小，人均水平低，外向度不高，实现跨越式发展的压力与挑战巨大；经济结构性矛盾仍很突出，特色优势产业规模较小，新兴产业发育不足，工业化、城镇化、农业产业化进程较慢；城乡基础设施尚未得到根本改善，资源、环境与人口压力日益增大；社会事业发展滞后，公共服务水平较低；城乡居民收入渠道单一，就业再就业形势依然严峻；社会管理中仍面临一系列新情况、新问题，维护稳定的任务十分艰巨；发展环境还不优，政府工作水平和效率还有待进一步提高；少数干部的群众观念淡薄，工作方法简单，个别干部存在腐败现象等。对以上问题，我们一定高度重视，采取有效措施，认真加以解决，绝不辜负全市人民的殷切希望，以出色的业绩报答全市人民的信赖和支持！
　　 
　　 二、未来五年，加速崛起、兴亳富民
　　 
　　 站在“十二五”这个新的起点上，我们肩负重任，倍感使命光荣、机遇扑面、前程似锦。“十二五”时期，是我市全面建设小康社会的关键期，是新型工业化、城镇化的快速推进期，是奋力赶超、加速崛起的黄金发展期，我们面临着前所未有的难得机遇。国际国内经济结构调整和产业转移加速推进，我国工业化、信息化、城镇化、市场化、国际化深入发展，为我市承接产业转移创造了更多有利条件；国家坚持扩大内需战略、大力推进中部地区崛起，省委、省政府支持皖北地区加快发展、开展南北结对合作，为我市加速崛起提供了强有力的政策支持；建市十年来积蓄的力量开始释放，全市上下加快发展的认识更加统一，思路更加清晰，措施更加有力。只要我们顺应人民群众的新期待，立足科学发展的新实践，就一定能够再创佳绩、再创辉煌！
　　 按照《中共亳州市委关于制定国民经济和社会发展第十二个五年规划的建议》，“十二五”时期我市经济社会发展的指导思想是：深入贯彻落实科学发展观，坚持“发展为上、民生为先、稳定为重，少说多做、真抓实干”，以科学发展为主题，以优化经济结构、加快转变发展方式为主线，以确保快速发展、力求争先进位为主调，牢牢抓住产业转移、加快皖北发展的重大战略机遇，坚持工业化、城镇化双轮驱动，推动开放发展、跨越发展、和谐发展，加速崛起进程，为全面建成小康社会奠定更加坚实的基础。
　　 市委《建议》要求，到“十二五”末，经济总量突破1000亿元，经济发展速度和居民收入赶上或超过全省平均水平，财政收入、全社会固定资产投资、人均地区生产总值三项指标翻一番以上；综合实力加速提升，经济结构不断优化，改革开放深入推进，居民收入较快增加，社会建设明显加强，社会更加和谐稳定。
　　 根据市委《建议》，市政府编制了《亳州市国民经济和社会发展第十二个五年规划纲要（草案）》。综合考虑，今后五年的主要奋斗目标是：生产总值年均增长13%以上，财政收入年均增长20%，社会消费品零售总额年均增长16%，全社会固定资产投资年均增长26%，外贸进出口总额年均增长20%以上，城镇居民人均可支配收入、农民人均纯收入年均分别增长13%以上。
　　 实现上述目标，必须重点做好以下九个方面的工作：
　　 （一）以新型工业化为主导，着力提升产业竞争水平。坚持工业强市不动摇，立足现有产业基础和资源优势，壮大主导产业，做强特色产业，提升传统产业，培育新兴产业。大力实施产业发展规划，延伸产业链条，加快建设年销售收入1000亿元的现代中药产业基地、500亿元的白酒及农产品加工基地、400亿元的煤电煤化工基地，加快建设皖北汽车及零部件生产基地和电子、纺织、服装鞋帽等以劳动密集型产业为主的加工制造业基地，积极发展节能照明、生物医药、新能源、新材料、新能源汽车等新兴产业，努力在产业聚集上取得突破。深入实施“125”工业振兴计划，培育龙头企业，到“十二五”末，培育发展年销售收入2000万元以上的规模工业企业1000家，形成2个年销售收入500亿元以上的产业集群，建设5个年产值100亿元以上的产业园区，力争规模以上工业企业个数、实现增加值、完成投资在2010年基础上翻两番，努力在产业和企业规模上取得突破。加大园区建设力度，每个省级开发区每年扩区3平方公里，力争到“十二五”末，单个开发区面积达到20平方公里；大力推进乡镇工业功能区和农民返乡创业园建设，每个建制镇建设一个2平方公里的工业功能区，努力在工业发展平台建设上取得突破。
　　 （二）以新型城镇化为重点，着力提升城镇化水平。把加快推进新型城镇化作为“十二五”跨越发展的强大动力，科学制定城镇发展规划，力争未来五年城镇化率提高10个百分点，主城区和3个县城的人口、面积均扩展50%以上。按照区域性中心城市的定位，规划建设亳州中心城区，大力推进南部新区建设，启动建设涡北新城，提升西部工业园区，加快老城区改造，推进道东片区开发。到2015年，中心城区人口达到60万人以上，建成区面积达到60平方公里，为2030年建成100万人口的现代化大城市打下坚实基础。按照远期50万人口的现代化中等城市发展目标，规划建设三县县城，力争“十二五”时期新区基本建成、初步展示形象。按照建设微小城市的要求，规划建设一批中心镇、特色镇，提高城镇产业、要素集聚能力和就业吸纳能力。按照“科学规划、农民自愿、及时复垦”的原则，尽快完成新村规划，抓好土地整治整村推进工作，完善新村配套设施，推进“村村通”工程建设。切实加强城镇管理，抓好文明城镇创建工作，治理脏、乱、差。“十二五”期间，中心城区要争创国家级园林城市、国家级文明创建工作先进城市，三县县城要争创省级文明县城、省级园林县城，各乡镇要争创市级文明城镇。
　　 （三）统筹城乡发展，着力提升农业现代化水平。把解决好农业、农村、农民问题作为全市工作的重中之重，着力在农业产业化、农村社区化、农民现代化三个方面取得重大突破。坚持以农民增收为核心，以实施百亿斤粮仓、百万亩中药材、百家重点产业化龙头企业、百万农村劳动力转移“四百工程”为载体，推动农业升级转型，到2015年，全市粮食总产稳定在100亿斤以上，中药材种植面积发展到100万亩以上，亿元以上农业产业化龙头企业发展到100家，转移农村劳动力100万人以上；创建国家级现代农业示范区1-2个，市级以上现代农业示范区30个，国家级、省级标准化养殖示范场30个；全市有效灌溉面积达到643万亩，旱涝保收田面积达到536万亩，农业机械总动力达到850万千瓦，森林覆盖率达到21%。
　　 （四）扩大消费需求，着力提升服务业发展水平。依托名城、名人、名胜、名产资源，打造“天下道源、曹魏故里、中华药都、养生亳州”旅游品牌，加快建设文化旅游和养生保健基地。加强商业网点建设，发展各类专业市场，完善城乡流通体系，加快培育一批年销售额超亿元的商贸流通企业。大力开拓农村市场，积极促进农民消费。合理引导汽车、文化、体育健身等新兴消费，支持发展电子商务、连锁经营、物流配送等新型业态，加快培育金融保险、信息中介、休闲娱乐、广告会展等现代服务业。
　　 （五）加强基础设施建设，着力增强保障发展能力。把交通建设放在突出位置，进一步完善交通规划和布局，全面建成泗许高速公路亳州段工程，积极推进济祁高速公路、亳宿铁路、商杭客运专线亳州段工程建设，争取307省道改造工程尽快开工，加快绕城快速通道建设，积极推进亳州机场前期工作，提高涡河、茨淮新河航运通航能力，力争通过“十二五”时期的努力，基本建成功能健全、安全高效的综合交通运输体系。争取实施“引淮入亳”工程，大力推进骨干河流治理工程，全面加强中小河流治理、农村饮水安全工程建设，完成中型涵闸除险加固工程，进一步改善农业生产条件。加快推进电力、供水、污水处理等城乡配套基础设施建设。加强数字亳州建设，实现“三网融合”，提升信息化应用水平。
　　 （六）推进生态文明建设，着力增强持续发展能力。合理开发和利用自然资源，落实节能减排目标责任制，加强环保执法工作，大力发展循环经济和低碳经济，建设资源节约型和环境友好型社会。加强农田防护林体系建设，提高城市绿地覆盖率，争创省级生态示范市、示范县和国家级、省级环境优美乡镇。加大对采煤塌陷区综合整治力度，实施煤矿环境保护与治理工程。推进城乡水环境治理，开展农村集中水源地保护工作，确保饮用水优质安全。
　　 （七）坚持科教兴市和人才强市战略，着力增强创新发展能力。完善技术创新体系，大力利用高新技术、先进适用技术改造提升传统产业。抓好中药产业研发创新能力建设，着力培育中药制造高新技术企业和高新技术产品。巩固提高义务教育质量和水平，加快普及高中阶段教育，加快发展高等教育，逐步普及学前教育，大力发展职业教育，重视发展特殊教育和民族教育，积极发展继续教育，鼓励扶持民办教育。积极筹建亳州学院，加快建设华佗技师学院。实施《亳州市中长期人才发展规划纲要》，启动科技创新人才等十大人才培养工程，促进各类人才脱颖而出。
　　 （八）加强社会建设，着力增强和谐发展能力。坚持民生为先，加大民生工程投入，促进基本公共服务均等化。大力促进创业带动就业，统筹做好各类群体就业工作。完善社会保障体系，扩大基本养老、基本医疗及失业、工伤、生育保险覆盖面。着力抓好城乡社会救助体系、社会福利和慈善事业建设。加快建设农村敬老院，全市五保老人集中供养率达到70%。深化医药卫生体制改革，稳妥推进公立医院改革，鼓励社会资本以多种形式兴办医疗机构，加强医疗服务体系和医疗保障体系建设，强化卫生监督执法、疾病预防控制和妇幼保健工作，提高公共卫生服务能力，大力发展中医药事业。稳定低生育水平，遏制出生人口性别比偏高势头，提高出生人口素质。大力实施文化强市战略，繁荣文化事业，发展文化产业。加强文化体育场馆建设，开展全民健身运动，提高竞技体育水平。高度重视民族工作，加强民族团结。
　　 切实加强社会管理，实施“六五”普法规划，加强人民调解工作，提高全民法律素质。严格落实信访责任制，继续坚持领导定期接访和带案下访制度，着力排查化解矛盾纠纷。加强应急管理，提高对突发公共事件的处置能力。扎实做好安全生产工作，坚决遏制重特大事故发生。深入开展平安亳州建设，健全社会治安防控体系，依法打击各类违法犯罪活动，努力创造和谐稳定的社会环境。依法规范宗教活动，坚决打击各类邪教组织。认真做好国防教育、国防动员和民兵预备役工作，支持驻亳部队建设，开展军民共建活动，巩固发展军政军民团结。
　　 （九）深化体制机制改革，着力增强开放发展能力。扎实推进事业单位人事制度改革，建立完善绩效考核机制。继续深化国有企业改革，支持企业在资本市场融资，培育民营企业上市。抓好金融改革，优化金融环境。坚持和完善农村基本经营制度，抓好土地流转平台建设，探索进城农民承包土地经营权和宅基地使用权有偿流转、有偿退出机制。加大招商引资力度，深化与央企对接、与民企对接、与芜湖结对合作，密切与周边地区、友好城市之间的经贸交流。坚持“引进来”和“走出去”相结合，积极筹建海关和保税物流园，提高外向型经济发展水平。大力支持全民创业，鼓励发展非公有制经济。着力提升政府工作效能，全面加强“信用亳州”建设，打造诚信药都。进一步规范市场行为，营造良好发展环境。
　　 
　　 三、2011年，提升标杆、争先进位
　　 
　　 2011年，是实施“十二五”规划的开局之年，也是新一届政府任期的第一年。我们将按照市委的统一部署，坚持发展为上不动摇、民生为先不懈怠、稳定为重不折腾，少说多做、真抓实干，提升发展标杆，力求争先进位，确保开好局、起好步，力争各项工作在全省争先进、在皖北创一流，奋力推进亳州大发展、大建设、大跨越。
　　 今年全市经济社会发展的主要预期目标是：生产总值增长13%以上；财政收入增长20%以上；固定资产投资增长40%以上；社会消费品零售总额增长17%以上；外贸进出口总额增长20%以上；利用外商直接投资增长30%，利用内资增长40%；城镇居民人均可支配收入、农民人均纯收入增长10%以上；人口自然增长率控制在8。5‰以内；居民消费价格指数控制在3。5%左右。
　　 （一）集中力量抓好项目建设和招商引资工作。抢抓政策机遇，继续开展“项目建设年”活动，严格落实项目工作责任制，坚持实行“月调度、季考查、分级管理、重点帮扶”的工作推进机制；建立健全项目谋划滚动机制，积极做好项目前期和对接争取工作，力争有更多项目列入国家和省投资计划，尽快启动实施一批列入市“十二五”规划的重大项目。年内实施2000万元以上续建项目138个、完成投资105亿元，新开工项目128个、完成投资100亿元，全年新谋划项目不少于800个、总投资2000亿元以上。
　　 全力保障泗许高速公路亳州段建成通车，促进济祁高速公路亳州段开工；推进307省道一级公路和202省道改造工程前期工作，改造县乡公路200公里；积极推动商杭高铁、禹亳铁路、亳宿铁路前期工作；加快涡河航道疏浚工程前期工作，争取列入国家建设补助计划；开工建设涡河涡阳三桥、蒙城三桥。优化亳州电网结构，实施伯阳500千伏变电站一期工程，新建、扩建一批220千伏、110千伏输变电工程，加快实施农网升级工程。建成利辛至亳州天然气管道工程，实现向涡阳县城、亳州城区及沿线部分重点镇供气，推进安庆至亳州成品油长输管道（亳州段）工程前期工作。强化项目建设管理，确保项目建设质量。
　　 继续开展“招商引资年”活动，坚持把招商引资作为第一要事，按照招大引强、优势互补、互利共赢的工作思路，进一步完善优惠政策，落实推进机制，加大激励力度，优化招商环境，创新招商方式，拓宽招商渠道。新组建若干招商中心，围绕五大主导产业、劳动密集型产业、现代服务业和新兴产业，着力引进一批带动作用强的大项目、好项目，全年实现招商引资400亿元以上。
　　 （二）全面促进工业结构转型升级。坚持工业强市不动摇，一手抓优势产业扩张，一手抓新兴产业培育，一手抓扶优扶强，一手抓靠大挂强，以工业的超常规发展带动全市经济社会的快速发展。年内，完成工业投资150亿元，增长50%；规模工业增加值增长28%以上；新增规模工业企业150家，新增亿元企业15家。
　　 加快建设现代中药产业基地，大力培育龙头企业和产业集群，全市药业企业达到120家以上，中药加工业产值达到90亿元。加强煤炭资源开发利用，加快建设利辛板集煤矿、涡阳涡北循环经济园，积极筹建利辛板集坑口电厂，推进蒙城赵集煤矿、涡阳信湖和花沟煤矿前期工作，煤炭生产能力增加到700万吨；配合做好铁矿、石油等矿产资源的勘察工作。依托粮食精深加工、畜牧水产果蔬加工、林木产品加工龙头企业，延伸产业链条，壮大产业规模，全市农产品加工产值达到220亿元以上。加快推进蒙城江淮安驰微型车项目建设，积极发展利辛江淮扬天等汽车改装和专用车辆生产，配套发展涡阳财富康达等汽车零部件生产，实现整车年生产能力1万辆，汽车和零部件制造业年产值25亿元。大力扶持谯城区杉杉服装等劳动密集型产业，筹建南部新区劳动密集型产业园。扶持新兴产业发展，抓好国能蒙城生物发电、利辛天极之光绿色照明、天利太阳能组件项目建设，发展新型墙体材料和彩印包装制品。
　　 大力实施古井集团、双轮集团跨越式发展战略，进一步扩大主导产品的市场份额。支持引导中小企业与大企业协作配套，突出抓好民营企业发展，促其不断发展壮大。推动企业上市融资，实现1家企业上报申报材料、2家进入上市辅导期的目标。深入推进质量兴市战略，新增省级企业技术中心2家、安徽名牌产品4个、安徽省著名商标10件。大力发展低碳经济，落实节能减排责任制，全面完成年度目标任务。
　　 加大园区扩容升级力度，增加投入，扩大规模，做强产业。抓好省级开发区建设，确保5个省级开发区再扩区15平方公里。加强与结对市县、省属大企业的合作，共同建设区中园、园中园。加快乡镇工业功能区建设，支持扩权试点镇申报筹建省级开发园区。在产业发展上，重点规划建设现代中药、煤炭化工、白酒及农副食品、汽车及零部件、现代物流、服装加工、筛网纱门产业园等一批特色产业园区，突出抓好古井白酒产业园、芜湖亳州工业园建设。
　　 （三）进一步加强城市建设和管理。围绕“中华药都、养生亳州”的城市定位，着眼建设百万人口城市，充分发挥规划的龙头作用，加快编制各类规划，完善规划管理体系。通过多种形式筹措资金，计划在中心城区年度投资50亿元，重点加快南部新区建设，推动老城区片点改造，启动涡河城区段沿岸开发，着力打造城市综合体、文教园区、华佗国际中药城等城市核心区。做好建安路涡河大桥和105国道涡河大桥前期工作。加强城市管理，巩固园林城市、文明城市创建成果，加大行政执法管理力度，坚决制止私搭乱建行为。今年，在中心城区重点实施以下10大工程：（1）开工建设投资48亿元的市南部新区4个安置还原小区；（2）实施投资9亿元的宋汤河西岸刘庄棚户区改造工程；（3）完成投资6亿元的市南部新区工业组团扩区5平方公里任务；（4）开工建设投资2。1亿元的南部新区市政公园；（5）实施投资6000万元的南部新区污水处理厂一期工程；（6）实施投资1。3亿元的老城区坑塘治理一期工程；（7）实施涡河沿岸街区改造一期工程；（8）实施投资2300万元的曹操地下运兵道出口、南京巷钱庄改造工程；（9）实施投资2亿元的华佗技师学院一期工程；（10）完成投资3亿元的亳州市人民医院新院一期工程。
　　 按照“新区拉框架、老区增功能、整体上品位”的思路，加快三县县城建设，完善垃圾处理、污水处理和各类管网等市政公用设施。大力加强乡镇建设，每县区确定3—5个基础较好、人口较多的集镇，按照3—5万人规模先行规划建设。开展文明乡镇创建工作，各乡镇逐步新建或完善一条街、一个农贸市场、一个居住小区；试点镇和重点扶持镇启动建设一个工业小区、一个污水处理厂、一个垃圾处理场，大力改善集镇生产生活环境，促进农民进镇安居乐业。
　　 （四）积极推动农业和农村经济发展。实施百亿斤粮仓建设工程，认真落实强农惠农政策，积极争取上级支持，组织开展小麦高产攻关、玉米振兴计划和粮油高产高效优质创建活动，确保粮食增产2%以上。实施特色农业开发工程，重点扶持药业企业发展基地，加强对中药材专业合作社和种植大户的引导，稳步扩大全市中药材种植面积。继续推进蔬菜产业提升行动，力争新增设施蔬菜5万亩。进一步加快标准化规模养殖场和养殖小区建设，强化科技服务和疫病防控，力争肉、蛋、奶总产增长6%以上，新建、扩建40个标准化规模养殖场（小区），规模养殖比重达到55%。实施农业产业化提升工程，积极探索农村土地规模经营新方式，力争全市土地流转面积达到100万亩、农民专业合作社总数发展到1600家。实施现代农业示范区创建工程，加快谯城十八里现代中药园区和大杨粮食加工示范区、涡阳义门农副产品加工集聚区、利辛粮食科技园和循环经济示范园区建设，力争创建市级农业产业化示范区2—3个、省级农业产业化示范区1个。
　　 实施农业生产保障工程，全面提高骨干河流防洪标准，加大中小河流治理力度，扎实推进沿淮重点平原洼地治理、灌区续建改造和泵站更新改造，完成赵王河、利阚河治理及西淝河洼地治理工程，启动油河、洺河、武家河、赵王河上段治理和20座病险涵闸除险加固工程。继续推进农村饮水安全工程，新解决40万人饮水安全问题。加快建设区域性农业科研中心，推广农作物优良品种和高产高效栽培技术。大力实施防灾减灾监测预警工程和粮食增产气象服务工程。加强农田林网和绿色长廊建设，建设新村环村林带。进一步提高农业装备水平。切实加强基本农田保护，全面启动千村土地整治工程，有序推进撤并自然村、建设中心村工作。大力推进农村社区综合服务体系建设，实施“清洁乡村美化家园”工程。
　　 （五）着力提高服务业发展层次。科学编制商业发展规划，加快以康美（亳州）华佗国际中药城、天运物流园为重点的物流园区建设，推进信旺集团商业综合体、家乐福超市、沃尔玛广场、春雨光彩汽车城等重点商贸项目建设，加快中国（亳州）中药材交易中心、亳州农产品批发市场改造升级，支持亳州百大、利辛粮油商厦、涡阳新华电、蒙城五洲华联等龙头企业做大做强，加快发展连锁经营、特许经营、电子商务、物流配送、便利店、仓储店等新型业态。继续推进“万村千乡市场工程”和“新网工程”，新建和改造农家店200家。大力实施“家电下乡工程”、“摩托车下乡工程”、“家电以旧换新工程”、“家政服务工程”，积极开展“农超对接”活动，进一步规范繁荣城乡市场。办好2011年国际（亳州）中医药博览会暨第27届全国（亳州）中药材交易会、2011年中国（亳州）酒文化节，积极发展会展经济。大力促进对外贸易，实现进出口总额2。5亿美元。
　　 继续实施旅游业发展三年行动计划，加强旅游基础设施建设，筹建中华药都养生园、文化旅游娱乐园，培育精品线路，开展宣传促销，全年旅游门票收入突破600万元，旅游总收入达到40亿元。引导各银行业金融机构创新金融产品，强化金融服务，努力扩大有效信贷投放，力争新增贷款80亿元以上。大力引进异地股份制银行来亳设立分支机构，加快徽商银行机构建设，支持芜湖扬子农村商业银行和淮南通商农村合作银行在我市设立村镇银行。大力培育小额信贷组织，新设立小额贷款公司5家以上。
　　 （六）大力发展各项社会事业。全面实施教育改革和发展规划纲要，加大教育投入，整合教育资源，完善教育督导制度，促进教育优先发展；启动学前教育3年行动计划，建设一批公办幼儿园；完成标准化学校建设任务，促进义务教育均衡发展；普及发展高中教育，推进省级优秀示 示范高中创建工作；启动亳州师专迎评和亳州学院筹建工作，继续加强亳州职业技术学院内涵建设，推进与安徽中医学院合作办学，提升高等教育办学水平；鼓励发展民办教育，规范社会力量办学行为；规划建设市职教园区、县职教中心，大力发展职业教育；加强教师队伍建设，实施名师培养计划和校长素质提升计划；推进教研科研工作，提高教育教学质量；完成校舍安全工程建设任务，净化校园周边环境，创建安全文明校园。积极推进科技创新，实施2-3个重大高新技术成果产业化项目，培育2个创新型企业，组建10个市级以上优势科技创新团队，力争全年高新技术产业增加值占GDP的比重提高到5%以上。做好高层次人才的培养、引进和使用工作，加强人力资源市场建设，启动建设人才楼和白领、蓝领公寓。
　　 全面深化医药卫生体制改革和基层医改，突出抓好重大传染病防控，实现公共卫生服务均等化；切实加强医疗服务监管和卫生监督执法工作，抓好乡镇卫生院标准化建设和文明卫生创建活动，启动市人民医院创建三甲医院工作；加快市精神病医院、谯城区人民医院等卫生项目建设，鼓励社会资本兴办医疗机构，大力发展中医药事业。加强文化强市建设，巩固改革成果，完善基础设施，筹建文化产业园，建设市图书馆、档案馆和五位一体的文化艺术中心；深入开展“扫黄打非”、网吧治理专项行动，规范文化市场秩序。开展全民健身运动，加强体育基础设施建设，培养体育后备人才。抓好人口和计划生育工作，稳定低生育水平。加强国防后备力量建设，深入做好征兵、“双拥”和转业退伍军人安置工作，进一步巩固军民团结。积极发挥工会、共青团、妇联等人民团体的重要作用。加强统计基层基础工作。做好国税、地税、工商、质监、人事、编制、档案、地方志、广播电视、新闻出版工作，推进外事、侨务、对台、港澳事务、人防、地震、气象、科协、老龄、红十字会和无线电管理工作，发展残疾人事业和慈善事业。
　　 （七）继续做好保障和改善民生工作。加强组织协调，细化任务分解，落实配套资金，全力实施33项民生工程，确保完成年度任务。多渠道拓展就业岗位，统筹做好各类群体就业工作，全市城镇新增就业2。64万人，新增转移农村劳动力5。6万人，城镇登记失业率控制在4。2%以内。认真抓好新型农村养老保险试点工作，积极开展城镇居民基本养老保险工作，不断扩大社会保险覆盖面，努力健全城乡统筹的社会保障体系。逐步完善社会救助体系，加强城乡低保规范化管理，加快社会化养老建设步伐，提高五保供养水平，集中供养率达到42%。优化土地资源配置，严格做到依法和谐征迁，年内储备报批土地1。8万亩。加快保障性住房建设，新建廉租房6200套、公共租赁房2200套。加强物价监管，坚决查处销售假、冒、伪、劣产品行为。
　　 （八）全力维护社会和谐稳定。启动实施“六五”普法规划，全面推进依法治市进程。深入开展平安创建活动，加强社会治安防控体系建设，深入开展打黑除恶、命案侦破、网上追逃等专项行动，严厉打击各种违法犯罪行为。认真落实信访工作责任制，扎实开展矛盾纠纷排查化解活动，切实维护社会大局稳定。加强突发事件应急处置能力建设，提高应急救援水平。全面落实安全生产责任制，深入开展重点行业和领域的安全整治行动，严防各类安全事故发生。加强食品药品监督管理工作，保障人民群众饮食用药安全。切实加强民族团结，依法管理宗教事务。
　　 
　　 四、努力建设人民满意的政府
　　 
　　 政府的一切权力来自于人民，人民满意、人民幸福是我们施政的终极目标。全市各级政府和政府全体工作人员，深入贯彻落实科学发展观，把人民群众的期盼作为政府工作的第一信号，把人民群众的满意作为政府工作的第一追求，始于群众需要，终于群众满意，努力建设人民满意的政府。
　　 （一）依法行政，建设法治政府。依法行政是政府工作的基本准则。我们必须严格依照法定权限管理经济社会事务，落实行政执法责任制，强化行政复议和执法监督，确保公正文明执法。进一步清理行政许可项目，规范行政许可程序，加强行政许可监督。坚持依法、科学、民主决策，规范行政决策程序，构建公众参与、专家咨询论证和政府决定相结合的行政决策体制。自觉接受市人大及其常委会的法律监督，接受市政协的民主监督，认真办理人大代表议案、建议和政协委员提案，虚心听取和采纳民主党派、工商联、无党派人士的意见。接受新闻舆论监督和社会公众监督，加强监察、审计等内部监督，确保权力在阳光下运行。推进政务公开，坚持网络问政，办好市长热线和市民论坛，欢迎群众监督政府。凡政府出台的政策、决定的事项，必须立说立行，落实到位，切实维护政府的公信力。
　　 （二）服务人民，建设高效政府。服务人民是政府的天职。我们必须牢固树立群众观点，创新服务群众的方式，以公仆的情怀、真诚的态度、细致的工作，赢得人民群众的拥护和支持。必须下大气力加强机关效能建设，优化政务环境，提高服务水平。认真落实为民、为企服务全程代理制度，全面推行马上办、限时办、联合办、网上办、公开办等行之有效的服务方式，为人民群众提供更加便捷的优质服务。进一步精简公文数量，精简会务活动，腾出更多的精力抓发展、抓大事。进一步建立健全政府工作落实机制，实行“一线工作法”，深入基层，调查研究，对重点工作实行分管副市长周调度、市长月调度、遇到问题及时解决的机制，真正形成一级抓一级、一级对一级负责、一级支持一级、一级维护一级的工作氛围。进一步建立健全政府绩效考评体系，实行压力传递，做到既定的目标任务层层分解到部门、落实到具体责任人，每项工作都有部署、有检查、有奖惩。继续开展民主评议百名科长和模拟仿真督查机关效能活动，重点解决少数干部精神不振、不负责任、推诿扯皮、执行不力的问题。
　　 （三）锐意进取，建设实干政府。新的形势和任务，对政府工作提出了更高要求，我们必须抢抓机遇，奋勇争先，少说多做，真抓实干。要坚定发展信心，咬住发展目标，以新思维谋划新发展，以新举措取得新突破，在困难中大干，在大干中赶超，在赶超中崛起。要把原则性和灵活性结合起来，敢于坚持原则，善于攻坚克难。要始终保持昂扬向上的精神状态、干事创业的工作激情，对党充满忠诚，对人民充满感情，对事业充满责任，敢于先行先试，做到实干苦干，把勇气、智慧、信心和力量，转化为兴亳富民的扎实行动，努力干出经得起实践、人民、历史检验的实绩。
　　 （四）从严治政，建设廉洁政府。廉洁是从政的底线，是为人立身之本。越是大建设、大投入，越要加强廉政建设。我们必须严格落实党风廉政建设责任制和领导干部廉洁从政的各项规定，坚持用制度管权、用制度管事、用制度管人，加强惩治和预防腐败体系建设，坚决纠正损害群众利益的不正之风，坚决整治破坏发展环境的突出问题。重点抓好工程建设领域专项治理，严格落实工程建设招投标、经营性土地招拍挂、政府采购等制度，切实保证资金安全、工程安全、生产安全、干部安全。政府每一个工作人员都要警钟长鸣，珍惜岗位，珍惜名节，堂堂正正做人，清清白白做事。
　　 各位代表！
　　 “长风破浪会有时，直挂云帆济沧海”。回顾“十一五”光辉历程，我们豪情满怀；踏上“十二五”新的征途，我们信心百倍！亳州美好的未来在呼唤着我们！让我们在中共亳州市委的坚强领导下，紧紧团结和依靠全市人民，万众一心，开拓奋进，为实现科学发展、加速崛起、兴亳富民的宏伟目标而努力奋斗！
